# Genetic Risk Factors of Neurodegeneration: The Role of *MMP1* rs1799750 (−1607 1G/2G), *MMP2* [rs243865 (−1306 C > T), rs2285053 (−735 C > T), and *MMP3* rs3025058 (−1171 5A/6A) Genetic Variants in Multiple Sclerosis Pathogenesis

**DOI:** 10.3390/jcm15197648

**Published:** 2026-10-02

**Authors:** Arzu Ay, Nevra Alkanli, Sezgin Kehaya, Ayse Nur Cesme

**Affiliations:** 1 Department of Biophysics, Faculty of Medicine, Trakya University, Edirne 22030, Turkey; anurcesme@icloud.com; 2 Department of Biophysics, Faculty of Medicine, Haliç University, Istanbul 34060, Turkey; nevraalkanli@halic.edu.tr; 3 Department of Neurology, Faculty of Medicine, Trakya University, Edirne 22030, Turkey; sezginkehaya@trakya.edu.tr

**Keywords:** multiple sclerosis, matrix metalloproteinases, gene variants, neurodegeneration, therapeutic targets

## Abstract

**Background/Objectives**: Matrix metalloproteinases (MMPs) are enzymes that play a key role in the disruption of blood–brain barrier integrity and neuroinflammatory processes. The objective of this study is to elucidate the contribution of Matrix Metalloproteinase 1 (*MMP1*) rs1799750 (−1607 1G/2G), Matrix Metalloproteinase 2 (*MMP2*) [rs243865 (−1306 C > T), rs2285053 (−735 C > T)], and Matrix Metalloproteinase 3 (*MMP3*) rs3025058 (−1171 5A/6A) genetic variants to the pathogenesis of multiple sclerosis (MS), and to evaluate their potential as genetic markers of neurodegeneration in conjunction with clinical and environmental factors. **Methods**: The study included 100 MS patients and 100 healthy controls. Genotype distributions of *MMP1*, *MMP2*, and *MMP3* genetic variants were determined using Polymerase Chain Reaction (PCR) and Restriction Fragment Length Polymorphism (RFLP) methods. Participants’ demographic data and clinical histories were recorded. The relevant *MMP* loci were genotyped, and genotype–allele frequencies as well as haplotype combinations were evaluated. Their associations with clinical parameters were analyzed using Chi-square tests, Odds Ratios (ORs), and One-Way Analysis of Variance (One-Way ANOVA). **Results:** The rates of heart disease history (13% vs. 4%, *p* = 0.031), alcohol consumption (29% vs. 14%, *p* = 0.011), and smoking (35% vs. 21%, *p* = 0.029) were significantly higher in MS patients compared to the control group. The *MMP2* rs243865 (−1306) TT genotype showed a strong positive association with MS, exhibiting approximately 10-fold higher odds of the disease (*p* < 0.001). Similarly, the *MMP3* rs3025058 5A/6A genotype was associated with 2.45-fold higher odds of MS (*p* < 0.003). Higher odds of the disease were particularly evident with the *MMP2* rs243865 (−1306) TT and *MMP3* rs3025058 6A/6A combination (OR: 6.769, *p* < 0.001), followed by the *MMP2* rs243865 (−1306) TT and *MMP2* rs2285053 (−735) CT combination (OR: 7.169, *p* < 0.001). No statistically significant association was found between the examined genotypes or haplotypes and clinical parameters, including attack frequency and disease duration (*p* > 0.05). **Conclusions:** Our study indicates that *MMP2* and *MMP3* genetic variants are significantly associated with MS susceptibility, though these variants do not appear to directly influence disease severity parameters such as attack frequency and disease duration. These findings further support the potential involvement of MMPs, particularly in the immunopathological phase of the disease. Consequently, addressing modifiable lifestyle factors in individuals carrying susceptibility-associated genetic profiles and considering early interventions targeting MMP pathways may offer valuable strategic insights for personalized MS management. The data obtained suggest that *MMP* variations have a potential to be not merely disease markers, but also early-stage therapeutic markers that may be addressed before the first clinical attack occurs. The present study also emphasizes the necessity of transitioning from a reactive approach to a proactive, genetically based personalized medicine paradigm in MS management.

## 1. Introduction

Multiple sclerosis (MS) is a chronic, autoimmune, and neurodegenerative disease of the central nervous system and is one of the leading causes of non-traumatic neurological disability among young adults worldwide [1]. The prevalence of this disease, which affects a significant number of people worldwide, varies by ethnic origin [2]. The pathogenesis and prognosis of MS are complex and not yet fully understood [3]. Pathogenetically, it is thought that MS may result from an interaction between genetic predisposition and environmental triggers [2]. More than half of the inheritance of MS is explained by the interaction between genetic, gene–environment, and gene–epigenome factors [4]. The underlying pathophysiology of the disease is characterized by a process in which immune cells activated in the periphery—due to genetic predisposition and the influence of environmental triggers—cross the blood–brain barrier, leading to inflammation, demyelination, and axonal damage in the central nervous system [1,5].

In people with MS, the course of the disease can range from a highly active and rapidly worsening condition to a benign and long-term condition. It is important to identify an effective treatment that can alter the course of the disease. For this reason, in addition to early and accurate diagnosis of the disease, it is also necessary to identify risk factors that could lead to a poor prognosis. Diagnostic and prognostic biomarkers derived from biological samples collected from patients with MS are needed for effective treatment management [6]. The identification of clinical and genetic biomarkers for MS will provide information about the diagnosis, activity, progression, and response to treatment of the disease [7,8,9,10].

Matrix metalloproteinases (MMPs) belong to the metalloproteinase family and play a key role in the degradation of the extracellular matrix [11]. In addition, MMPs play a role in pathological processes such as inflammation through the degradation of the basement membrane [12]. MMPs are secreted, and membrane-bound enzymes and are classified as stromelysins, collagenases, gelatinases, matrilysins, enamelins, and membrane-type MMPs [13].

Studies have shown that MMPs play a key role in regulating the dynamic structure of the extracellular matrix during immune cell infiltration and tissue destruction processes [13]. MMPs, a family of zinc-dependent endopeptidases, have the ability to break down components of the basement membrane and the extracellular matrix. In the pathogenesis of MS, MMPs play an active role in processes such as increased blood–brain barrier permeability, the activation of pro-inflammatory cytokines, and the direct destruction of the myelin sheath [12,13]. Gelatinases such as MMP-2, in particular, are of interest as disease biomarkers because they disrupt the integrity of the blood–brain barrier, thereby facilitating the passage of inflammatory cells into the brain parenchyma [14]. Individual susceptibility to MS and the rate of disease progression may be regulated by single-nucleotide variants in the promoter regions of *MMP* genes. Functional variants such as *MMP1* rs1799750 (−1607 1G/2G) and *MMP3* rs3025058 (−1171 5A/6A) cause differences in enzyme levels by altering the gene’s transcriptional activity [3]. In addition, it has been shown that *MMP2* variants [rs243865 (−1306 C > T) and rs2285053 (−735 C > T)] influence susceptibility to MS either on their own or in synergy with other *MMP* genes [14].

The aim of this study is to determine the distribution of *MMP1*, *MMP2*, and *MMP3* gene variants in multiple sclerosis (MS) patients, to compare this genetic profile with that of healthy individuals in order to contribute population-specific data to the literature, and to analyze the correlation between these genetic variants and the clinical course of the disease. Ultimately, the findings are expected to contribute to the prediction of MS susceptibility and the development of molecular-based strategies for prognostic evaluations.

## 2. Materials and Methods

### 2.1. Standardization of MS and Healthy Control Groups

This study was submitted to the Ethics Committee for Non-Interventional Clinical Research at Trakya University Faculty of Medicine, and ethical approval was obtained under protocol number TÜTF-GOBAEK 2023/147. We also confirm that all methods used in our study are fully compliant with the guidelines and regulations of the Institutional Ethics Committee. This study was conducted in accordance with the guidelines of the Helsinki Declaration. Signed informed consent forms were obtained from a group of patients diagnosed with multiple sclerosis and from a healthy control group. In determining the sample size for this study, the meta-analysis by Mohammadhosayni and colleagues titled “Gene Polymorphisms in the Matrix Metalloproteinase (MMP) Family and the Risk of Multiple Sclerosis: A Systematic Review and Meta-Analysis” was used as the primary reference [12]. Based on a power analysis conducted with a 5% margin of error and 95% power, it was determined that a total of 130 participants—with at least 65 in each group—would be sufficient. In this context, our study was conducted with a group of 200 participants, consisting of 100 people with MS and 100 healthy volunteers. The participants (both patients and controls) were recruited from the selected population residing in the Thrace region of Turkey. Patients were diagnosed based on clinical symptoms, cerebrospinal fluid tests, and magnetic resonance imaging. Our study did not include individuals with any neurodegenerative or neuroinflammatory diseases affecting the central nervous system other than MS, those with other significant autoimmune disorders that could directly affect MMP expression, or individuals diagnosed with any malignancy. Information regarding specific disease-modifying therapies (DMTs) used by individual MS patients at the time of sample collection was not systematically available in this cohort. Consequently, treatment status could not be included as a covariate in the statistical models, which represents a limitation of our study. Routine peripheral blood samples collected in tubes containing ethylenediaminetetraacetic acid (EDTA) were used to determine the genotype distribution of *MMP* gene variants among the participants.

### 2.2. Deoxyribonucleic Acid (DNA) Isolation and Quality Control

Genomic DNA was extracted from EDTA-containing blood samples collected from the patient and control groups using a commercial DNA isolation kit (Invitrogen, Carlsbad, CA, USA). This process was repeated for samples found to be insufficient in terms of quantity or quality following isolation. The purity and concentration of the isolated DNA samples were measured using a Nanodrop spectrophotometer (NanoDrop Technologies, Wilmington, DE, USA), and sample integrity was verified by 0.8% agarose gel electrophoresis.

### 2.3. The PCR and RFLP Analyses

PCR amplifications were performed using a TECHNE thermal cycler (Techne, Staffordshire, UK). Following amplification, the PCR products were digested using restriction enzymes (Thermo Scientific, Waltham, MA, USA) according to the manufacturer’s instructions, and the resulting fragments were analyzed by PCR-RFLP assay. A 25 µL PCR mixture was prepared containing 50 ng of template DNA, 0.2 mmol/L of each deoxynucleoside triphosphate (dNTP), as well as forward and reverse primers (Invitrogen, Carlsbad, CA, USA), 1× PCR buffer, 3 mM MgCl_2_, and 1.25 units of Taq DNA polymerase (Fermentas-Invitrogen, Vilnius, Lithuania). The primer sequences used in the analyses are as follows:

*MMP1* rs1799750 (−1607 1G/2G): 5′-TCGTGAGAATGTCTTCCCATT-3′ (Forward), 5′-TCTTGGATTGATTTGAGATAAGTCATATC-3′ (Reverse);
*MMP2* rs243865 (−1306 C > T): 5′-CTTCCTAGGCTGGTCCTTACTGA-3′ (Forward) 5′-CTGAGACCTGAAGACCTAAAGAGCT-3′ (Reverse);
*MMP2* rs2285053 (−735 C > T): 5′-GGATTCTTGGCTTGGCGCAGGA-3′ (Forward) 5′-GGGGGCTGGGTAAAATGAGGCTG-3′ (Reverse);
*MMP3* rs3025058 (−1171 5A/6A): 5′-CTTCCTGGAATTCACATCACTGCCACCACT-3′ (Forward) 5′-GGTTCTCCATTCCTTTGATGGGGGGAAAAA-3′ (Reverse).

Specific thermal cycling protocols were applied for each gene region. Detailed cycle times and temperatures are provided in Supplemental File S1. Annealing temperatures were optimized to prevent the formation of nonspecific products. As part of the RFLP method, PCR products were incubated at 37 °C for 3 h using appropriate restriction enzymes (*PdmI*, *BfaI*, *HinfI*, *PsyI*) and 1x Buffer Tango. Enzymatic hydrolysis processes have been experimentally optimized in terms of buffer composition, enzyme concentration, and duration. PCR products were visualized using 2.0% agarose gel electrophoresis, while the products used to determine genotype distributions were visualized using 3.0% agarose gel electrophoresis. To ensure the reliability of the analyses, positive and negative controls were used at every stage. The identified genotypes and band lengths are as follows:

*MMP1* rs1799750 (−1607 1G/2G): Targets the insertion/deletion region at position −1607. Successful amplification resulted in an 113-bp fragment that was cleaved into 1G or 2G alleles using the *PdmI (XmnI)* restriction enzyme.
*MMP1* rs1799750 (−1607 1G/2G):
1G/1G homozygous (102 bp, 11 bp), 1G/2G heterozygous (113 bp, 102 bp, 11 bp), 2G/2G homozygous (113 bp).
*MMP2* [rs243865 (−1306 C > T) and −735 C > T)]: Two different promoter regions of the *MMP2* gene were genotyped. The −1306 C > T region (188 bp fragment) was analyzed via *BfaI* digestion, while the −735 C > T region (391 bp fragment) was analyzed via *HinfI* digestion.
*MMP2* rs243865 (−1306 C > T):
TT homozygous (162 bp, 26 bp, 5 bp), CT heterozygous (188 bp, 162 bp, 26 bp, 5 bp), CC homozygous (188 bp, 5 bp).
*MMP2* rs2285053 (−735 C > T): TT homozygous (338 bp, 53 bp), CT heterozygous (391 bp, 338 bp, 53 bp), CC homozygous (391 bp).
*MMP3* rs3025058 (−1171 5A/6A): The adenine variant at position −1171 was amplified into a 129-bp product and genotyped using the *PsyI (Tth111I)* enzyme.
*MMP3* rs3025058 (−1171 5A/6A):
5A/5A homozygous (96 bp), 5A/6A heterozygous (129 bp, 96 bp), 6A/6A homozygous (129 bp).

**Figure 1:**
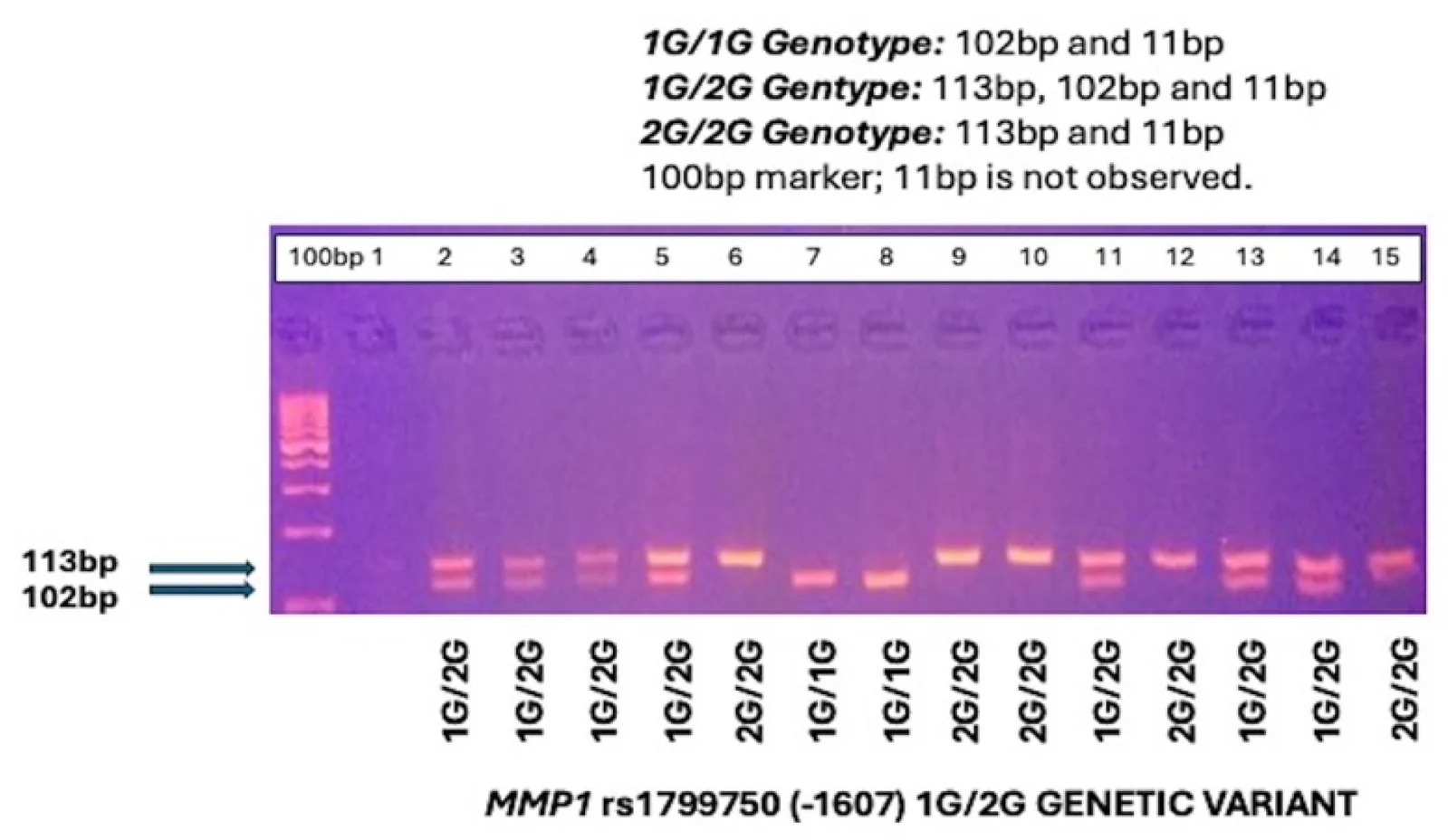
*MMP1* rs1799750 gene variation genotype distribution.

**Figure 2:**
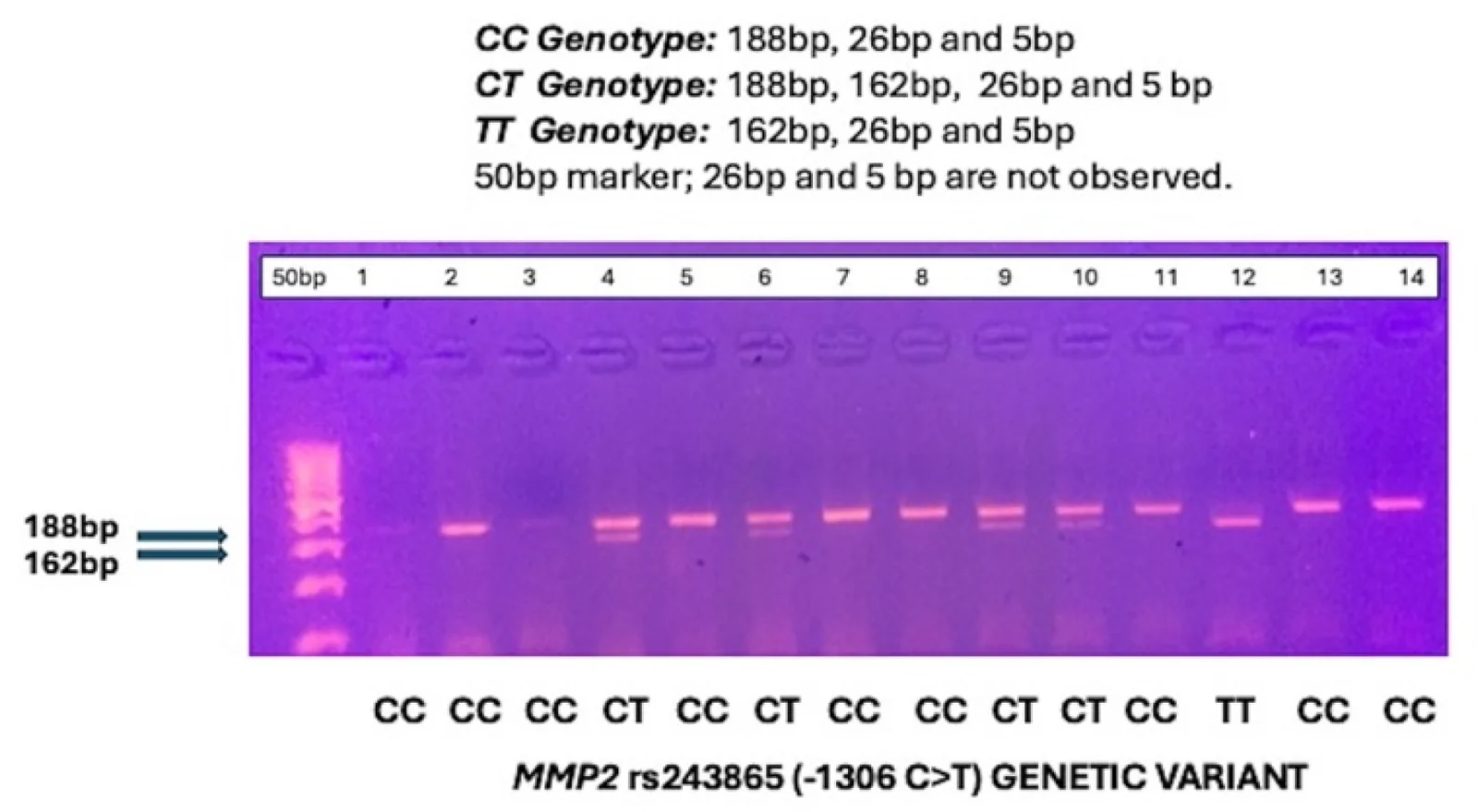
MMP2 rs243865 gene variation genotype distribution.

**Figure 3:**
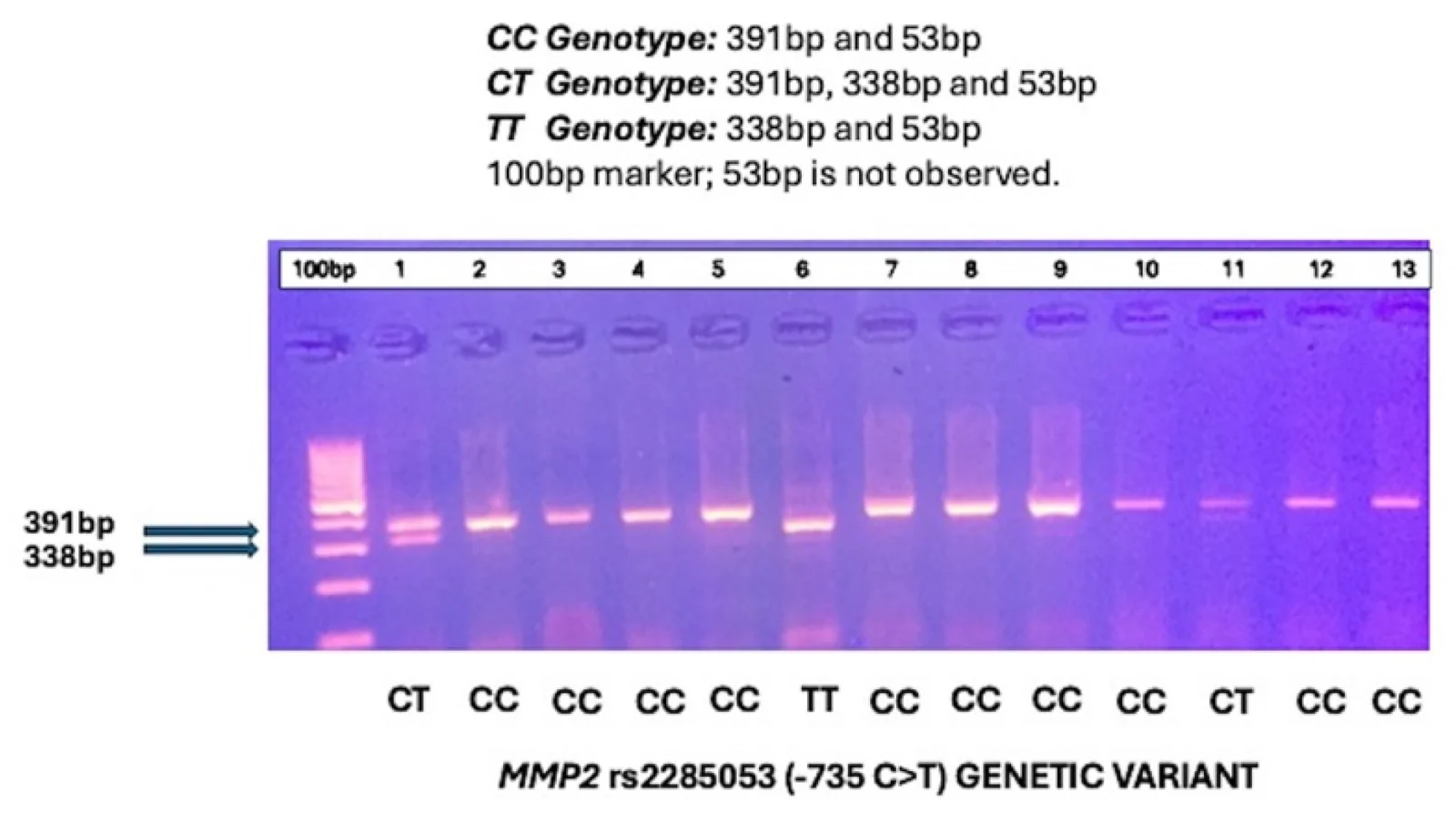
MMP2 rs2285053 gene variation genotype distribution.

**Figure 4:**
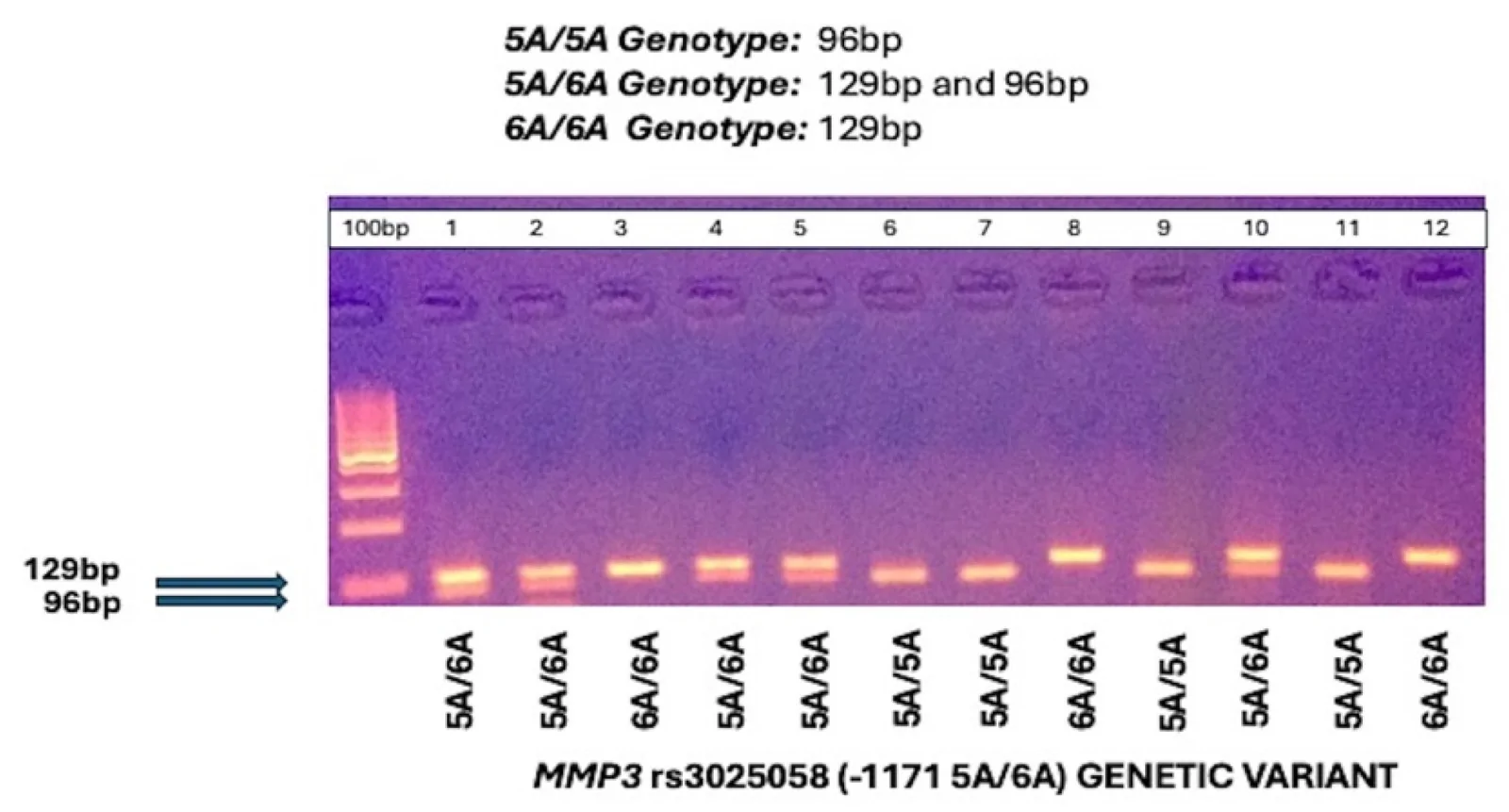
MMP3 rs3025058 gene variation genotype distribution.

Primer sequences (forward and reverse), specific PCR cycle conditions (denaturation and annealing temperatures, and number of cycles), and the fragment lengths obtained for each genotype (e.g., 1G/1G, 1G/2G, 2G/2G) are provided in Supplementary Files S1 and S2 (Figure 1, Figure 2, Figure 3 and Figure 4).

### 2.4. Statistical Analysis

The statistical analysis of the data obtained from the study was conducted using SPSS (Statistical Package for the Social Sciences) software (Version 25, IBM Corp., Armonk, NY, USA). Continuous variables (age, mean number of attacks, duration of MS) are expressed as mean ± standard deviation (SD). Categorical variables (genotype frequencies, tobacco/alcohol use, comorbidities) are presented as counts and percentages (n, %). To compare the demographic and clinical characteristics between the patient and control groups, either the independent samples *t*-test or the chi-square (χ2) test was used, depending on the nature of the variable. Demographic characteristics, including age and sex composition, were compared between the patient and control groups using independent samples *t*-tests and chi-square tests, respectively. Furthermore, a Principal Component Analysis (PCA) was performed on standardized variables [age, sex, Type 2 diabetes mellitus (Type 2 DM), hypertension, cholesterol, heart disease, smoking, and alcohol use] to assess the underlying dimensional structure and interrelationships among comorbidities, lifestyle habits, and demographic parameters, ensuring the robustness of our findings against potential confounding effects. The distribution of genotype frequencies for *MMP1*, *MMP2*, and *MMP3* gene variants across the groups was analyzed using the chi-square test. Haplotype analysis was performed to examine the co-inheritance patterns of these genetic variants. The effect of gene combinations (*MMP1*/*MMP2*, *MMP1*/*MMP3*, *MMP2*/*MMP3*) on MS susceptibility was calculated separately for each combination. Confidence interval (CI) values were calculated to determine the relative risk of specific genotypes, haplotypes, and clinical risk factors in the development of MS. In the analysis of the relationships between genotypes and haplotype combinations and clinical parameters (average number of attacks and duration of the disease), differences between groups were assessed using one-way ANOVA. To evaluate the incremental predictive utility of the investigated gene variants beyond traditional clinical and demographic factors (age, sex, hypertension, Type 2 DM, cholesterol, heart disease, alcohol, and smoking), Nagelkerke R2 values were calculated from binary logistic regression models. The baseline clinical model yielded a Nagelkerke R2 of 0.082. The inclusion of the genetic variants improved the model’s explanatory power: the Nagelkerke R2 increased to 0.095 for *MMP1* (−1607 1G/2G), 0.284 for *MMP2* (−1306 C > T), 0.112 for *MMP2* (−735 C > T), and 0.128 for *MMP3* (−1171 5A/6A). Notably, the *MMP2* (−1306 C > T) variant demonstrated a substantial improvement in predictive performance, underscoring its potential clinical utility as a biomarker for MS. In all statistical comparisons, a *p*-value of < 0.05 was considered statistically significant. The specific *p*-values obtained in the analyses are listed in the relevant sections of the tables.

## 3. Results

### 3.1. Demographic and Clinical Characteristics of the Study Population

No statistically significant differences were observed between the MS patient group (n = 100) and the healthy control group (n = 100) regarding age (47.610 ± 12.542 vs. 48.130 ± 8.810 years, respectively; *p* = 0.735) or the prevalence of basic comorbidities such as hypertension (*p* = 0.852), Type 2 DM (*p* = 0.818), and hypercholesterolemia (*p* = 0.253). However, analysis of lifestyle and clinical characteristics revealed notable associations with the patient group. Specifically, the prevalence of heart disease was 13% in patients compared to 4% in controls (OR: 3.586, 95% CI: 1.127–11.413, *p* = 0.031). Furthermore, higher frequencies were observed for alcohol consumption (29% vs. 14%; OR: 2.509, *p* = 0.011) and cigarette smoking (35% vs. 21%; OR: 2.026, *p* = 0.029) in the MS cohort. These findings point to a notable coexistence of cardiovascular and lifestyle-related factors within this patient population (Supplemental File S3). Both groups showed no significant differences regarding age and sex. To further determine the relationship between comorbidities, smoking, cholesterol, age, and sex, a PCA was conducted. The first two principal components accounted for 39.6% of the total variance. The PCA loadings revealed that metabolic and age-related variables (hypertension, age, Type 2 DM, and cholesterol) formed an independent cluster, while lifestyle and clinical outcomes (alcohol use, heart disease, and smoking) separated distinctly. This independent grouping confirms that the elevated risk observed in the patient group is not driven by demographic or general lifestyle confounders, reinforcing its association with the genetic background.

### 3.2. Comparison of MMP Genotypes Between Patients and Controls

The distribution of variants in the *MMP1*, *MMP2*, and *MMP3* genes was examined between the MS patient group (n = 100) and the healthy control group (n = 100) included in the study, and the data obtained were analyzed comparatively. The *MMP2* rs243865 (−1306 C > T) genotype distribution showed a statistically highly significant difference between the patient and control groups (*p* < 0.001). The prevalence of the TT genotype was 53% in the patient group, while it was only 10% in the control group. Statistical analysis revealed that the TT genotype was significantly associated with higher odds of developing MS, showing approximately 10-fold higher odds compared to the control group (OR: 10.149, 95% CI: 4.736–21.751, *p* < 0.001. On the contrary, the CC genotype was significantly more prevalent in the control group (37%) than in the patient group (6%), indicating a protective effect or lower odds of MS (OR: 0.109, *p* < 0.001). The distribution of *MMP3* genotypes showed a significant difference between groups (*p* < 0.012). The heterozygous 5A/6A genotype was found to be significantly higher in the patient group (45%) compared to the control group (25%). This genotype was significantly associated with higher odds of MS, showing approximately a 2.45-fold increase (OR: 2.455, 95% CI: 1.347–4.473, *p* < 0.003). The 6A/6A genotype, observed more frequently in the control group (57%), was found to be negatively associated with MS (OR: 0.546, *p* = 0.035). There was no statistically significant difference in *MMP1* genotype distributions between the patient and control groups (*p* = 0.531). The OR values calculated for each of the three genotypes (1G/1G, 1G/2G, 2G/2G) did not reach statistical significance. The genotype distribution difference for the *MMP2* rs2285053 (−735) locus remained at the statistical threshold but did not reach significance (*p* = 0.097). Although the TT genotype was slightly more common in the patient group (7% vs. 2%), this difference was not statistically significant (*p* = 0.109) (Table 1).

### 3.3. Haplotype Analysis and Distribution Between Patients and Controls

The haplotype distribution of *MMP* genes between MS patients and control groups was analyzed, and numerous significant combinations associated with susceptibility or protection against the disease were identified. Biparental haplotype analyses conducted between the MS patient group and healthy controls have revealed the effect of co-inheritance of genetic variants on disease risk. In the combined evaluation of the *MMP1* and *MMP2* rs243865 (−1306 C > T) loci, haplotype combinations containing the TT genotype showed a significant positive association with higher odds of MS. The 1G/1G-TT (OR: 5.630, *p* = 0.003) and 1G/2G-TT (OR: 5.051, *p* = 0.002) haplotype combinations were found to be significantly higher in the patient group compared to the control group. In contrast, combinations containing the CC genotype (1G/1G-CC, 1G/2G-CC, and 2G/2G-CC) were determined to be associated with protection against the disease and were observed more frequently in the control group (*p* < 0.05). *MMP1*/*MMP2* rs2285053 (−735 C > T). No statistically significant difference was found between the groups in the haplotype analysis. (*p* > 0.05). In *MMP1*/*MMP3* haplotype analysis, the 1G/2G-5A/6A combination was significantly more frequent in the patient group (21%) than in the control group (9%), showing a positive association with a 2.68-fold increase in the odds of MS (*p* = 0.021). The 2G/2G-5A/5A combination was more pronounced in the control group and showed a protective effect (OR: 0.165, *p* = 0.021). The combined analysis of two different *MMP2* loci revealed the strongest positive associations and highest odds of the disease. The TT-CC (OR: 6.093, *p* < 0.001) and TT-CT (OR: 7.169, *p* < 0.001) haplotype combinations were observed at a significantly high frequency in the MS patient group. In contrast, CC-CC, CC-CT, and CT-CC combinations were more prevalent in the control group, indicating a protective effect and lower odds of MS (*p* < 0.01). The highest odds of susceptibility to the disease were detected in the TT-6A/6A (OR: 6.769, *p* < 0.001) and TT-5A/6A (OR: 3.917, *p* = 0.005) combinations in the *MMP2* rs243865 (−1306 C > T)/*MMP3* haplotype analysis. In the control group, the highest protective effects were determined in the CC-6A/6A (OR: 0.104, *p* < 0.001) and CT-5A/5A (OR: 0.137, *p* = 0.010) combinations (Table 2).

### 3.4. Relationship Between MMP Genotypes and Clinical Attack Frequency

The relationship between *MMP1*, *MMP2*, and *MMP3* gene variants and the mean number of clinical attacks were analyzed. No statistically significant differences were observed in attack frequencies across the different genotypes of the *MMP1* rs1799750 (−1607 1G/2G), *MMP2* [rs243865 (−1306 C > T) and rs2285053 (−735 C > T)], and *MMP3* loci (all *p* > 0.05). Although a slight clinical trend was noted among *MMP3* genotypes, it did not reach statistical significance (*p* = 0.126). Detailed genotype-specific attack counts are provided in Supplemental File S4.

### 3.5. Association Between MMP Haplotype Pairs and Clinical Attack Frequency

The relationship between different MMP gene locus haplotype pairs and the mean number of clinical attacks in MS patients was also evaluated. Consistent with the individual locus analyses, no statistically significant associations were identified between any of the examined haplotype combinations—including *MMP1*/*MMP2*, *MMP1*/*MMP3*, *MMP2*/*MMP2*, and *MMP2*/*MMP3* pairs—and clinical attack frequencies (all *p* > 0.05). Detailed haplotype-specific attack counts and subgroup distributions are presented in Supplemental File S5.

### 3.6. Association Between MMP Genotypes and Disease Duration

Similarly, the comparison between the genotype distributions of the MS patients and their average disease duration revealed no statistically significant relationships across any of the examined *MMP1*, *MMP2*, and *MMP3* genetic loci (all *p* > 0.05). Although minor numerical differences in disease duration were observed among various genotypes, none of these variants reached statistical significance. Detailed genotype-specific disease durations are provided in Supplemental File S6.

### 3.7. Association Between MMP Haplotype Pairs and Disease Duration

The effects of the co-inheritance of different *MMP* gene loci (haplotypes) on the duration of MS disease were also examined. Statistical analyses revealed no significant relationships between any examined haplotype combinations—including *MMP1*/*MMP2*, *MMP1*/*MMP3*, and *MMP2*/*MMP3* pairs—and disease duration (all *p* > 0.05). Although numerical differences were noted across certain subgroups, these variants lacked statistical significance, often due to limited subgroup sizes. Detailed haplotype-specific disease durations are provided in Supplemental File S7.

### 3.8. Incremental Predictive Value of Genetic Variants (Nagelkerke R2 Analysis)

To evaluate whether the investigated gene variants add meaningful predictive power and clinical utility beyond traditional demographic and clinical factors, binary logistic regression models were constructed. The baseline clinical model—incorporating age, sex, hypertension, Type 2 DM, cholesterol status, heart disease, alcohol consumption, and smoking status—yielded a Nagelkerke R2 of 0.082.

When individual genetic variants were added to the baseline model, varying degrees of improvement in model performance were observed (Supplemental File S8). The inclusion of the *MMP1* (−1607 1G/2G) variant slightly increased the Nagelkerke R2 to 0.095 (ΔR2 = +0.013). Similarly, the addition of the *MMP2* (−735 C > T) and *MMP3* (−1171 5A/6A) variants resulted in modest improvements, with Nagelkerke R2 values of 0.112 (ΔR2 = +0.030) and 0.128 (ΔR2 = +0.046), respectively.

Notably, the incorporation of the *MMP2* (−1306 C > T) variant demonstrated a substantial enhancement in the model’s overall explanatory power, elevating the Nagelkerke R2 to 0.284 (ΔR2 = +0.202). This pronounced increase highlights the substantial incremental predictive value of the *MMP2* (−1306 C > T) variant, underscoring its potential as a robust genetic biomarker for multiple sclerosis status beyond classical risk factors.

## 4. Discussion

### 4.1. Demographic and Clinical Profile of the Study Cohort

The main results obtained were that the *MMP2* rs243865 (−1306) TT genotype and specific susceptibility-associated haplotype combinations (such as *MMP2* and *MMP3* interactions) are strongly associated with a substantially increased risk of MS. Furthermore, lifestyle factors including elevated smoking rates and increased alcohol consumption, alongside vascular comorbidities such as heart disease, emerged as critical clinical factors that parallel these genetic susceptibilities. Following these primary outcomes, each finding is discussed in detail below.

The absence of significant differences in key baseline characteristics—such as age, hypertension, and T2DM—demonstrates that the patient and control groups were well-matched, confirming that our study outcomes were not confounded by these demographic variables.

Heart disease emerged as a prominent clinical risk factor differentiating the patient group from the controls, demonstrating a high-risk magnitude among the evaluated parameters. In our study, the finding that heart disease is 3.5 times more common in people with MS indicates that vascular risk factors contribute to reduced cerebral reserve and increased lesion burden. The adverse effects of vascular comorbidities on the course of MS are increasingly emphasized in the literature [15]. The presence of vascular risk factors, such as heart disease, may increase the susceptibility of MS lesions to hypoxic damage by affecting cerebral blood flow and triggering chronic low-grade inflammation. This highlights the importance of addressing cardiovascular health alongside neuroinflammation in the comprehensive management of MS [16].

Regarding lifestyle factors, our findings indicate that the odds of smoking are substantially higher in the patient group compared to the control group, consistent with the literature showing that smoking is a strong risk factor for the development of MS. The components in cigarette smoke are thought to exert both pro-inflammatory effects and direct neurotoxic damage. Although smoking increases the permeability of the blood–brain barrier, facilitating immune cell infiltration into the central nervous system, substances in cigarette smoke—such as cyanide and nitric oxide—are believed to trigger axonal damage and accelerate myelin loss [17]. The high smoking rate of 35% observed in our cohort highlights how critical lifestyle modifications and smoking cessation counseling are in the clinical management of MS patients, as quitting smoking not only improves overall health but also has the potential to slow disease progression.

In addition, the rate of alcohol use was significantly higher in the patient group compared to the control group (29% vs. 14%). Alcohol’s ability to disrupt the blood–brain barrier and the pro-inflammatory processes it triggers through the gut microbiota may further negatively affect the pathogenesis of MS [1,18].

Moving to the genetic architecture, the *MMP2* rs243865 (−1306) TT genotype emerged as a factor strongly associated with higher odds of the disease, underscoring the importance of therapies that inhibit the expression of these enzymes. In this context, abnormal MMP concentrations—which have been widely documented to be detectable at different stages of MS [13,19,20,21]—further support the relevance of these pathways in disease progression. Conversely, the CC genotype of this variant—found to be significantly more common in the control group—suggests that this genotype may serve as a potential marker for protection. Furthermore, complex haplotype combinations (such as *MMP2* and *MMP3* rs3025058 interactions, including the 6A/6A or 5A/6A pairings) showed strong associations with higher odds of MS. Particularly in patients with these susceptibility-associated haplotypes, the presence of vascular comorbidities may accelerate central nervous system tissue destruction and lead to a more aggressive disease course. For instance, combinations like 1G/1G-TT or 1G/2G-TT likely promote more pronounced pro-inflammatory processes, while combinations like CC-CC, CC-CT, and CC-6A/6A are thought to associate with low proteolytic activity.

Ultimately, factors such as heart disease, smoking, and alcohol consumption, coupled with high-risk genetic variations, are significantly more prevalent in the MS patient group. This confirms that MS is not merely an isolated central nervous system disorder but is intimately linked to the patient’s overall vascular health and lifestyle profile [15,17,18,22]. Clinicians who adopt a multidisciplinary approach to the management of MS—encompassing cardiovascular screening, genetic risk profiling, and lifestyle modification—may play a pivotal role in optimizing the long-term prognosis of the disease.

### 4.2. Evaluation of MMP Variants as Genetic Susceptibility Factors for MS

Although regulated MMP activity contributes to physiological synaptic plasticity, unregulated MMP activity can lead to tissue damage. It has been determined that exogenous MMP-1 application in vitro stimulates dendritic branching. In vivo, however, an increase in dendritic complexity has been observed as a result of excessive MMP-1 expression. For this reason, it has been reported that MMP-1 plays a role in maintaining neuronal plasticity. Various MMPs, such as MMP-1, are thought to play an important role in processes such as neurogenesis, proliferation, and differentiation in neural stem cells. It has been found that reduced expression of MMPs associated with various *MMP* gene variants in the central nervous systems of patients with MS is linked to an insufficient capacity for tissue repair [13]. A previous study found that haplotypes containing the 6A allele of the *MMP3* rs3025058 (−1171 5A/6A) gene variant exhibit higher transcriptional activity during inflammation compared to haplotypes containing the 5A allele [23,24]. A study conducted among the Serbian population found no significant association between the *MMP3* rs3025058 (−1171 5A/6A) genetic variant and the development of MS. In another study, it was found that the mean central nervous system values in patients with relapsing-remitting MS who carried the 6A/6A homozygous genotype of the *MMP3* rs3025058 (−1171 5A/6A) gene variant were significantly higher than those in patients carrying the 5A allele [25,26,27]. In another study, it was found that the CC genotype and the C allele of the *MMP2* rs2285053 (−735 C > T) gene variant were significantly more common in patients with MS than in healthy controls. In addition, in the presence of the *MMP9* T, *MMP7* G, *MMP2* C (TGC) haplotype, it has been observed that this is associated with increased promoter activity and elevated protein expression of these MMPs. Consequently, it may increase the risk of MS by causing damage to the extracellular matrix and its associated components. It is believed that interactions among *MMP* genes also play an important role in susceptibility to MS [14].

The findings of our study suggest that variants in the *MMP* genes may play a decisive role in MS susceptibility. In particular, variants at the *MMP2* rs243865 (−1306 C > T) and *MMP3* rs3025058 (−1171 5A/6A) loci were found to be strongly associated with disease development. Our findings demonstrate that individuals with the TT genotype for the *MMP2* rs243865 (−1306 C > T) gene variant had a substantially higher risk of MS. The prevalence of the TT genotype in the patient group (10% versus 53%) suggests that this genetic variant may facilitate the chronic inflammatory response that triggers demyelination processes in the central nervous system. The markedly higher risk of MS observed in individuals with the *MMP2* rs243865 (−1306) TT genotype underscores the importance of therapies that inhibit the expression of these enzymes in the management of the disease. This genetic predisposition may serve as a pharmacogenetic marker that determines patients’ potential to respond to treatment or which medication should be preferred. Conversely, the CC genotype of this genetic variant—which was found to be significantly more common in the control group—suggests that this genotype may serve as a potential marker for protection against the disease. Our findings show that the heterozygous 5A/6A genotype for the *MMP3* rs3025058 (−1171 5A/6A) gene variant is significantly associated with an increased odds of MS. It is thought that elevated *MMP3* levels in individuals carrying the 5A allele may accelerate the activation of pro-inflammatory cytokines, thereby paving the way for increased axonal damage and lesion burden. The fact that the 6A/6A genotype, which was predominant in the control group (57%), is associated with lower odds of MS supports this hypothesis. In our study, no statistically significant association was found between the *MMP1* rs1799750 (−1607 1G/2G) and *MMP2* rs2285053 (−735 C > T) variants and MS (*p* > 0.05). This suggests that the loci in question do not have a significant effect on MS susceptibility in the studied population or that the effect of these genes is masked by other environmental and genetic factors. In our study, patients identified as having a high expression profile may benefit more from IFN-beta (IFN-β) treatment in terms of restoring the stability of the blood-brain barrier. For these patients, MMP inhibition is a key step in controlling neuroinflammatory exacerbations. Identifying genotypes with high MMP activity can be used to predict the rate of disease progression in patients [28].

### 4.3. Impact of MMP Haplotype Combinations on MS Susceptibility

The haplotype data obtained in our study indicate that enzymatic disruption in the pathogenesis of MS is shaped by the synergistic effects of multiple loci rather than a single genetic variant. Combinations of *MMP2* rs243865 (−1306 C > T) and *MMP3* rs3025058 (−1171 5A/6A) variants are particularly important for therapeutic intervention strategies. In the haplotype analysis, combinations of the *MMP2* TT genotype with either the *MMP3* rs3025058 6A/6A or the 5A/6A haplotypes were associated with significantly higher odds of MS. Patients with this high-risk haplotype profile may be more prone to damage to the blood–brain barrier. For these individuals, opting for disease-modifying therapies that are highly effective in the early stages of the disease may be a strategic step toward preventing neuroinflammatory damage. The TT-CT and TT-CC combinations, which showed the strongest association with the odds of MS, support the use of agents that directly inhibit these enzymes. Particularly in patients with haplotypes showing strong associations with MS, the use of MMP inhibitors such as minocycline in addition to standard treatments may reduce the frequency of attacks by preserving the integrity of the blood–brain barrier. Combinations such as CC-CC, CC-CT, and CC-6A/6A—which were significantly higher in the control group—are thought to be associated with low proteolytic activity. Patients with this profile are expected to respond better to standard first-line treatments and have a more stable course of the disease. On the contrary, pro-inflammatory processes are likely to be more pronounced in individuals with the 1G/1G-TT or 1G/2G-TT combinations. This finding may guide the physician to start with a more aggressive treatment rather than gradually increasing the treatment.

### 4.4. Gene-Environment Interactions in MS Pathogenesis

The clinical and genetic findings from our study indicate that the risk of MS is shaped not only by individual factors but also by the synergistic interaction of genetic predisposition and environmental triggers. Our study found that the smoking rate was significantly higher in the MS group, and the *MMP2* rs243865 (−1306) TT genotype emerged as a factor strongly associated with higher odds of the disease. In individuals with the *MMP2* rs243865 (−1306) TT genotype, the already genetically high enzyme expression potential, when combined with an environmental factor such as smoking, can lead to a significant increase in proteolytic stress on the blood–brain barrier. This finding suggests that individuals with a genetic predisposition may have a significantly higher risk of developing MS if they smoke. The prevalence of heart disease in our patient group was approximately 3.5 times higher than in the healthy control group. When evaluated in conjunction with our genetic findings, this suggests an interaction between the vascular and immune systems. It is known that chronic hypoxia and vascular inflammation associated with heart disease increase MMP-2 and MMP-3 activity [22]. In particular, the presence of vascular comorbidities in individuals with susceptibility-associated haplotype combinations—such as the TT-6A/6A or TT-5A/6A combinations—may accelerate tissue destruction in the central nervous system, leading to a more aggressive course of the disease. The significant increase in alcohol consumption in the MS group should be evaluated in the context of the susceptibility-associated haplotypes formed by the combination of the *MMP1* and *MMP3* genotypes (such as the 1G/2G-5A/6A combination). For individuals with genetic predispositions affecting matrix metalloproteinase pathways, avoiding modifiable risk factors such as smoking and alcohol consumption may serve as a critical preventive strategy. Furthermore, because vascular burdens heavily influence neuroinflammatory conditions, optimizing cardiovascular health not only enhances the overall quality of life in people with MS but may also help mitigate ongoing neuroinflammation.

### 4.5. Genetic Susceptibility Versus Clinical Parameters: Modifying Factors in MS

Although *MMP* gene variants are strong susceptibility factors for the development of MS, it is believed that clinical parameters such as attack frequency are shaped not only by genetic background but also by interactions with treatment adherence, environmental factors, and other modifier genes. While our haplotype analyses show that certain genetic combinations are significantly associated with higher odds of developing MS (by up to 7–10 times), no direct effect of these combinations on clinical course parameters, such as relapse frequency, has been detected. This finding suggests that MMP enzymes play a significant role in the disease-initiating (immune-triggering) phase, but that the frequency of flare-ups is primarily controlled by treatment response, lifestyle, and epigenetic factors.

Although the findings of our study indicate that *MMP* variants play a significant role in susceptibility to MS, no significant association was found between disease duration and genotypes. This finding indicates that while genetic susceptibility profiles determine the likelihood of developing the disease, the temporal progression (duration) of the disease is governed by far more complex variables, such as treatment regimens and individual biological responses. Our findings confirm that, although genetic variants in *MMP* largely determine susceptibility to MS, they have no direct effect on the temporal progression (duration) of the disease. The absence of a significant association between disease duration and genetic haplotypes suggests that MMPs may play a more prominent role in triggering the disease rather than in its progression. Although no statistically significant difference was found between disease duration and genotype distributions in MS, these data provide a critical foundation for determining long-term treatment strategies in patients with susceptibility-associated genetic profiles. The limited effect of *MMP* genotypes on disease duration suggests that these enzymes may be more effective at influencing the extent of tissue damage and the severity of the inflammatory response rather than the temporal progression of the disease. This situation underscores the need to take the genetic risk profile into account when selecting a treatment, regardless of the duration of treatment.

In individuals carrying susceptibility-associated haplotypes such as *MMP2* TT and *MMP3* rs3025058 5A/6A, the likelihood of blood–brain barrier damage is high, regardless of the duration of the disease. This situation may make the use of barrier-protective and MMP-inhibiting therapies in the early stages a therapeutic goal. Rather than predicting clinical course, haplotype analyses can help us understand the mechanism by which a patient developed MS. This situation may also make it easier to select targeted therapies based on the patient’s inflammatory burden.

Regardless of the duration of the disease, the potential for matrix degradation is significantly higher in patients carrying susceptibility-associated haplotypes such as *MMP2* rs243865 (−1306) TT and *MMP3* rs3025058 (−1171) 6A/6A. In these patients, in addition to standard treatments, the use of agents such as interferon-beta or minocycline—which have been shown to inhibit MMP enzymes—may be a strategic goal for preserving the integrity of the blood–brain barrier in the long term. In individuals with a short disease duration but who carry susceptibility-associated haplotypes, an early transition to more aggressive immunomodulatory therapies may minimize cumulative tissue damage. In our study, the fact that the group with the longest duration of illness has the 6A/6A genotype necessitates further investigation into the effects of these enzymes on the clearance of cellular debris and myelin repair during the chronic phase. In patients with chronic illness and a high MMP expression profile, adding neuroprotective agents to standard treatment may offer a new treatment option for slowing axonal loss. The fact that there is no significant difference between disease duration and genotype conveys to clinicians the message that “a patient newly diagnosed with the disease and a patient who has had MS for 15 years share the same genetic susceptibility background.” For this reason, monitoring the treatment response based not only on the year of diagnosis but also on serum MMP levels (as a biomarker) that are consistent with the patient’s genetic profile will enable the development of a personalized monitoring protocol. In conclusion, although our study did not find a direct association between *MMP* genotypes and the duration of MS or the frequency of relapses, our findings suggest that certain variants are significantly associated with higher odds of the disease. Taken together, these results point to the possibility that MMP enzyme systems may serve as critical therapeutic targets in the underlying pathophysiological processes (such as damage to the blood–brain barrier and the initiation of inflammation) rather than merely reflecting the clinical subtypes of MS. Nonetheless, further large-scale longitudinal studies are warranted to validate these preliminary insights.

### 4.6. Limitations of the Study

Although our study presents important findings regarding the role of *MMP* gene variants in MS, there are some limitations that should be taken into account when interpreting the results. Although our study, which included a total of 100 patients and 100 healthy controls, had sufficient statistical power for analyzing overall genotype distributions, the number of patients in the subgroups decreased significantly when the data were analyzed in relation to clinical parameters such as the number of attacks based on haplotype combinations. This situation may have made it difficult for certain numerical differences observed between clinical parameters and genetic variants to become statistically significant. The average number of attacks is influenced not only by genetic factors but also by external factors such as the type of immunomodulatory treatment patients receive, the timing of treatment initiation, and adherence to treatment. In our study, the moderating role of these variables on genotypic effects could not be fully isolated. The fact that the study was conducted at a single center and involved participants selected from a specific population may limit the generalizability of the findings to the general MS population. Multicenter studies involving diverse ethnic groups and larger cohorts will provide a clearer picture of the impact of the susceptibility-associated haplotype variants we have identified on clinical outcomes. Another important limitation of this study is the lack of detailed records regarding disease duration and the specific DMTs or immunosuppressant treatments used by the MS patients at the time of inclusion. Consequently, these clinical variables and treatment status could not be adjusted for in the statistical models, which should be considered when interpreting the results.

## 5. Conclusions

This study comprehensively examined the relationship between *MMP* gene variants and MS in terms of both genetic susceptibility and clinical parameters. The findings highlight the complementary roles of genetic predisposition and lifestyle factors in the pathogenesis of MS. Our findings suggest that MS is a systemic condition complicated by vascular risk factors and lifestyle variables, rather than an isolated neuroinflammatory process. In this context, to improve the long-term prognosis, it is essential that MS management evolve into a comprehensive, multidisciplinary strategy that includes cardiovascular screening and lifestyle changes, rather than being limited to neurological intervention alone. In patients with high-risk *MMP* genotypes, early initiation of treatments targeting matrix degradation or barrier repair is of great clinical importance. Close monitoring of smoking and vascular risk factors in individuals with a genetic predisposition should be a key strategy in preventing or delaying the onset of the disease. *MMP* gene variants are not only risk factors for MS but also serve as important molecular guides for understanding the pathophysiology of the disease and developing personalized treatment protocols. Identifying MMP-focused high-risk haplotypes can serve as a molecular guide for predicting the extent of blood–brain barrier damage in patients and for selecting targeted treatments that inhibit enzyme activity. In individuals with a genetic risk haplotype, measuring active enzyme levels in serum and cerebrospinal fluid is valuable for confirming the biochemical manifestation of genotypic predisposition. In patients with high-risk *MMP* variants, examining the relationship between blood–brain barrier permeability—as measured by magnetic resonance imaging—and lesion burden may shed light on the mechanism by which these enzymes contribute to tissue damage. Research is needed to determine whether the efficacy of drugs that affect MMP expression varies depending on the patient’s *MMP* genotype profile; this will enable the optimization of personalized treatment protocols. In conclusion, the *MMP* genetic variants identified in our study should be considered not only as susceptibility factors in the pathogenesis of MS but also as potential biomarkers for therapeutic interventions. In the future, evaluating the efficacy of drugs targeting these enzyme systems in individuals with high-risk *MMP* genotypes holds the potential to make a significant contribution to personalized medicine approaches in MS. These genotype and haplotype frequencies highlight the complexity of the genetic makeup of MS and the importance of individual risk profiles. MMP-focused identification of high-risk genotypes/haplotypes may serve as a molecular guide for predicting the extent of blood–brain barrier damage in patients and for selecting specific treatments that inhibit enzyme levels.

Although our sample size of 200 participants (100 MS patients and 100 healthy controls) was determined to be statistically sufficient based on a priori power calculations referencing prior meta-analytic data, this cohort size primarily provides adequate power to detect genetic variants with large effect sizes. Consequently, our study may be underpowered to identify variants with smaller or moderate effects. Therefore, while our findings offer valid preliminary insights, future multi-center replication studies involving larger cohorts (exceeding 1000 to 2000 individuals) are strictly required to robustly validate these genetic associations with multiple sclerosis across broader populations.

## Figures and Tables

**Table 1:** Logistic regression analysis and odds ratio values of *MMP* gene variants genotype distributions for multiple sclerosis patient and healthy control group.

*Genotype Distributions*		*Patient Group* *(n = 100)*	*Control Group* *(n = 100)*	*p*
***MMP1 rs1799750*** ***(−1607 1G/2G)***	1G/1G	35 (35.0%)	30 (30.0%)	0.531 ^a^
	1G/2G	39 (39.0%)	37 (37.0%)	
	2G/2G	26 (26.0%)	33 (33.0%)	
***MMP2 rs243865*** ***(−1306 C > T)***	CC	6 (6.0%)	37 (37.0%)	<0.001 ^a^*
	CT	41 (41.0%)	53 (53.0%)	
	TT	53 (53.0%)	10 (10.0%)	
***MMP2 rs2285053*** ***(−735 C > T)***	CC	52 (52.0%)	64 (64.0%)	0.097 ^a^*
	CT	41 (41.0%)	34 (34.0%)	
	TT	7 (7.0%)	2 (2.0%)	
***MMP3 rs3025058*** ***(−1171 5A/6A)***	5A/5A	13 (13.0%)	18 (18.0%)	<0.012 ^a^*
	5A/6A	45 (45.0%)	25 (25.0%)	
	6A/6A	42 (42.0%)	57 (57.0%)	
***Genotype distributions***		***Patient group (n = 100)*** ***and Control group (n = 100)*** ***OR (95%Cl)***		***p***
***MMP1 rs1799750*** ***(−1607 1G/2G)***	1G/1G	1.256 (0.694–2.274)		0.451 ^a^
	1G/2G	1.089 (0.615–1.927)		0.771 ^a^
	2G/2G	0.713 (0.387–1.314)		0.279 ^a^
***MMP2 rs243865*** ***(−1306 C > T)***	CC	0.109 (0.043–0.273)		<0.001 ^a^*
	CT	0.616 (0.352–1.078)		0.090 ^a^
	TT	10.149 (4.736–21.751)		<0.001 ^a^*
***MMP2 rs2285053*** ***(−735 C > T)***	CC	0.609 (0.346–1.074)		0.086 ^a^
	CT	1.349 (0.760–2.396)		0.307 ^a^
	TT	3.688 (0.747–18.211)		0.109 ^a^
***MMP3 rs3025058*** ***(−1171 5A/6A)***	5A/5A	0.681 (0.314–1.477)		0.330 ^a^
	5A/6A	2.455 (1.347–4.473)		<0.003 ^a^*
	6A/6A	0.546 (0.312–0.957)		<0.035 ^a^*

^a^ Logistic regression, Chi-square test; * Significance (*p* < 0.05). OR: Odds ratio; CI: Confidence interval; CC: Cytosine–Cytosine; CT: Cytosine–Thymine; TT: Thymine–Thymine; 1G/1G: 1Guanine/1Guanine; 1G/2G: 1Guanine/2Guanine; 2G/2G: 2Guanine/2Guanine; 5A/5A: 5Adenine/5Adenine; 5A/6A: 5Adenine/6Adenine; 6A/6A: 6Adenine/6Adenine. Key points in the table and significant *p*-values less than 0.05 are indicated in bold and italics.

**Table 2:** Haplotype analysis, frequency rates, and odds ratio values for *MMP* gene variants in patients with multiple sclerosis and healthy controls.

*Haplotype Analysis* *MMP1/MMP2 rs243865* *(−1306 C > T)*	*Patients* *(N = 100)*	*Frequency* *(%)*	*Controls* *(N = 100)*	*Frequency (%)*	*OR (95%Cl), p*
1G/1G-CC	3	***3.0***	15	***15.0***	*0.155 (0.043–0.554), **p*** **= *0.004*** *
1G/1G-CT	13	***13.0***	13	***13.0***	*1.000 (0.439–2.280), p* = 1.000
1G/1G-TT	19	***19.0***	4	***4.0***	5.630 (1.840–17.220), p = 0.003 *
1G/2G-CC	1	***1.0***	10	***10.0***	0.091 (0011–0.724), p = 0.024 *
1G/2G-CT	17	***17.0***	20	***20.0***	*0.819 (0.401–1.676), p* = 0.585
1G/2G-TT	21	***21.0***	5	***5.0***	5.051 (1.821–14.006), p = 0.002 *
2G/2G-CC	2	***2.0***	10	***10.0***	0.184 (0.039–0.861), p = 0.032 *
2G/2G-CT	11	***11.0***	18	***18.0***	*0.563 (0.251–1.263), p* = 0.164
2G/2G-TT	13	***13.0***	5	***5.0***	*2.839 (0.972–8.291), p* = 0.056
***Haplotype analysis*** ***MMP1/MMP2 rs2285053*** ***(−735 C > T)***	***Patients*** ***(N = 100)***	***Frequency*** ***(%)***	***Controls*** ***(N = 100)***	***Frequency (%)***	***OR (95%Cl), p***
1G/1G-CC	16	***16.0***	20	***20.0***	*0.762 (0.369–1.574), p* = 0.462
1G/1G-CT	18	***18.0***	10	***10.0***	*1.976 (0.862–4.526), p* = 0.107
1G/2G-CC	20	***20.0***	25	***25.0***	*0.750 (0.385–1.461), p* = 0.398
1G/2G-CT	16	***16.0***	13	***13.0***	*1.275 (0.578–2.811), p* = 0.548
2G/2G-CC	15	***15.0***	20	***20.0***	*0.800 (0.382–1.677), p* = 0.554
2G/2G-CT	8	***8.0***	10	***10.0***	*0.783 (0.296–2.073), p* = 0.622
2G/2G-TT	3	***3.0***	2	***2.0***	*1.516 (0.248–9.271), p* = 0.653
***Haplotype analysis*** ***MMP1/MMP3***	***Patients*** ***(N = 100)***	***Frequency*** ***(%)***	***Controls*** ***(N = 100)***	***Frequency (%)***	***OR (95%Cl), p***
1G/1G-5A/5A	7	***7.0***	4	***4.0***	*1.807 (0.512–6.376), p* = 0.358
1G/1G-5A/6A	13	***13.0***	12	***12.0***	*1.096 (0.474–2.535), p* = 0.831
1G/1G-6A/6A	15	***15.0***	13	***13.0***	*1.181 (0.530–2.630), p* = 0.684
1G/2G-5A/5A	3	***3.0***	5	***5.0***	*0.588 (0.137–2.528), p* = 0.475
1G/2G-5A/6A	21	***21.0***	9	***9.0***	2.688 (1.164–6.207), p = 0.021 *
1G/2G-6A/6A	16	***16.0***	23	***23.0***	*0.638 (0.314–1.296), p* = 0.214
2G/2G-5A/5A	2	***2.0***	11	***11.0***	0.165 (0.036–0.765), p = 0.021 *
2G/2G-5A/6A	13	***13.0***	6	***6.0***	*2.341 (0.852–6.430), p* = 0.099
2G/2G-6A/6A	10	***10.0***	17	***17.0***	*0.543 (0.235–1.252), p* = 0.152
***Haplotype analysis*** ***MMP2 rs243865*** ***(−1306 C > T)*** ***/MMP2 rs2285053*** ***(−735 C > T)***	***Patients*** ***(N = 100)***	***Frequency*** ***(%)***	***Controls*** ***(N = 100)***	***Frequency (%)***	***OR (95%Cl), p***
CC-CC	3	***3.0***	18	***18.0***	0.141 (0.040–0.495), p = 0.002 *
CC-CT	3	***3.0***	20	***3.0***	0.124 (0.036–0.431), p = 0.001 *
CT-CC	19	***19.0***	37	***37.0***	0.399 (0.210–0.760), p = 0.005 *
CT-CT	16	***16.0***	14	***14.0***	*1.170 (0.538–2.547), p* = 0.692
TT-CC	28	***28.0***	6	***6.0***	6.093 (2.395–15.498), p < 0.001 *
TT-CT	23	***23.0***	4	***4.0***	7.169 (2.378–21.608), p < 0.001 *
TT-TT	2	***2.0***	1	***1.0***	*2.020 (0.180–22.646), p* = 0.568
***Haplotype analysis*** ***MMP2 rs243865*** ***(−1306 C > T)*** ***/MMP3***	***Patients*** ***(N = 100)***	***Frequency*** ***(%)***	***Controls*** ***(N = 100)***	***Frequency (%)***	***OR (95%Cl), p***
CC-5A/5A	2	***2.0***	5	***5.0***	*0.388 (0.073–2.047), p* = 0.265
CC-5A/6A	2	***2.0***	9	***9.0***	0.206 (0.043–0.981), p = 0.047 *
CC-6A/6A	3	***3.0***	23	***23.0***	0.104 (0.030–0.358), p < 0.001 *
CT-5A/5A	2	***2.0***	13	***13.0***	0.137 (0.030–0.622), p = 0.010 *
CT-5A/6A	24	***24.0***	12	***12.0***	2.316 (1.085–4.941), p = 0.030 *
CT-6A/6A	16	***16.0***	28	***28.0***	0.490 (0.246–0.977), p = 0.043 *
TT-5A/6A	20	***20.0***	6	***6.0***	3.917 (1.500–10.227), p = 0.005 *
TT-6A/6A	22	***22.0***	4	***4.0***	6.769 (2.239–20.468), p < 0.001 *
***Haplotype analysis*** ***MMP2 rs2285053*** ***(−735 C > T)/MMP3***	***Patients*** ***(N = 100)***	***Frequency*** ***(%)***	***Controls*** ***(N = 100)***	***Frequency (%)***	***OR (95%Cl), p***
CC-5A/5A	5	***5.0***	11	***11.0***	*0.426 (0.142–1.274), p* = 0.127
CC-5A/6A	26	***26.0***	18	***18.0***	*1.601 (0.812–3.154), p* = 0.174
CC-6A/6A	21	***21.0***	35	***35.0***	0.494 (0.262–0.930), p = 0.029 *
CT-5A/5A	6	***6.0***	7	***7.0***	*0.848 (0.275–2.619), p* = 0.774
CT-5A/6A	17	***17.0***	8	***8.0***	*2.355 (0.966–5.743), p* = 0.060
CT-6A/6A	18	***18.0***	20	***20.0***	*0.878 (0.433–1.781), p* = 0.719
TT-5A/6A	3	***3.0***	1	***1.0***	*3.062 (0.313–29.949), p* = 0.336

OR: Odds ratio; CI: Confidence interval; * Significance (*p* < 0.05). CC: Cytosine–Cytosine; CT: Cytosine–Thymine; TT: Thymine–Thymine; 1G/1G: 1Guanine/1Guanine; 1G/2G: 1Guanine/2Guanine; 2G/2G: 2Guanine/2Guanine; 5A/5A: 5Adenine/5Adenine; 5A/6A: 5Adenine/6Adenine; 6A/6A: 6Adenine/6Adenine. Key points to be highlighted in the table and significant *p*-values less than 0.05 are indicated in bold and italics.

## Data Availability

The data presented in this study are available upon request from the corresponding author. The data are not publicly available due to ethical reasons.

## Supplementary Materials

The following supporting information can be downloaded at: https://www.mdpi.com/article/10.3390/jcm15197648/s1. Supplemental File S1: *MMP* gene variants primer series, PCR conditions and restriction enzyme; Supplemental File S2: Genotype distributions, PCR-RFLP components, product lengths for *MMP* gene variants; Supplemental File S3: Comparison of clinical and demographic parameters between patients with multiple sclerosis and healthy controls; Supplemental File S4: MMP gene variants genotype distributions according to average attack numbers for multiple sclerosis patient; Supplemental File S5: Haplotype analysis according to average attack numbers in patients with multiple sclerosis; Supplemental File S6: MMP gene variants genotype distributions according to MS duration for multiple sclerosis patient; Supplemental File S7: Haplotype analysis according to MS duration in patients with multiple sclerosis; Supplemental File S8: Model performance and Nagelkerke R2 change statistics.

## Abbreviations

The following abbreviations are used in this manuscript:

ANOVA	Analysis of Variance
CI	Confidence Interval
DMTs	Disease-Modifying Therapies
DNA	Deoxyribonucleic Acid
dNTP	Deoxynucleoside Triphosphate
EDTA	Ethylenediaminetetraacetic Acid
IFN-β	Interferon-beta
MMP-1	Matrix Metalloproteinase-1
MMP-2	Matrix Metalloproteinase-2
MMP-3	Matrix Metalloproteinase-3
MS	Multiple Sclerosis
OR	Odds Ratio
PCR	Polymerase Chain Reaction
RFLP	Restriction Fragment Length Polymorphism
SD	Standard Deviation
T2DM	Type 2 Diabetes Mellitus
