# Supplementary material for: Genetic Risk Factors of Neurodegeneration: The Role of MMP1 rs1799750 (−1607 1G/2G), MMP2 [rs243865 (−1306 C > T), rs2285053 (−735 C > T), and MMP3 rs3025058 (−1171 5A/6A) Genetic Variants in Multiple Sclerosis Pathogenesis
Source: J Clin Med. 2026 Oct 2;15(19):7648. doi: 10.3390/jcm15197648 (PMC13665806; doi:10.3390/jcm15197648)
Supplement: Supplementary file 1 [file jcm-15-07648-s001.zip › MS Supplemental File S1.pdf]

Supplemental File S1. *MMP* gene variants primer series, PCR conditions and restriction enzymes

Genetic Variants	*Primer series*	*PCR Conditions*	*RE*
MMP1 rs1799750 (-1607 1G/2G)	**FP:** 5′-TCGTGAGAATGTCTTCCCATT-3′<br>**RP:** 5′-TCTTGGATTGATTTGAGATAAGTCAT ATC-3′	5 minutes at 94⁰C<br>20 seconds at 94⁰C, 20 seconds at 55⁰C, 20 seconds at 72⁰C — 35cycle<br>5 minutes at 72⁰C	*PdmI (XmnI)*
MMP2 rs243865 (-1306 C>T)	**FP:** 5′-CTTCCTAGGCTGGTCCTTACTGA-3′<br>**RP:** 5′-CTGAGACCTGAAGACCTAAAGAGCT-3′	5 minutes at 94⁰C<br>45 seconds at 95⁰C, 45 seconds at 58⁰C, 45 seconds at 72⁰C — 35cycle<br>10 minutes at 72⁰C	*BfaI (XspI)*
MMP2 rs2285053 (-735 C>T)	**FP:** 5′-GGATTCTTGGCTTGGCGCAGGA-3′<br>**RP:** 5′-GGGGGCTGGGTAAAATGAGGCTG-3′	5 minutes at 94⁰C<br>45 seconds at 95⁰C, 45 seconds at 65⁰C, 45 seconds at 72⁰C — 35cycle<br>10 minutes at 72⁰C	*HinfI*
MMP3 rs3025058 (-1171 5A/6A)	**FP:** 5′-CTTCCTGGAATTCACATCACTGCCACCACT-3′<br>**RP:** 5'-GGTTCTCCATTCCTTTGATGGGGGGAAAAA-3'	3 minutes at 94⁰C<br>50 seconds at 94⁰C, 50 seconds at 58⁰C, 20 seconds at 72⁰C — 35cycle<br>5 minutes at 72⁰C	*PsyI (Tth111I)*

FP: Forward primer; **RP:** Reverse primer; **PCR:** Polymerase chain reaction; **RE:** Restriction enzyme
