# Supplementary material for: Genetic Risk Factors of Neurodegeneration: The Role of MMP1 rs1799750 (−1607 1G/2G), MMP2 [rs243865 (−1306 C > T), rs2285053 (−735 C > T), and MMP3 rs3025058 (−1171 5A/6A) Genetic Variants in Multiple Sclerosis Pathogenesis
Source: J Clin Med. 2026 Oct 2;15(19):7648. doi: 10.3390/jcm15197648 (PMC13665806; doi:10.3390/jcm15197648)
Supplement: Supplementary file 1 [file jcm-15-07648-s001.zip › MS Supplemental File S2.pdf]

Supplemental File S2: Genotype distributions, PCR-RFLP components, product lengths for *MMP* gene variants

Genetic Variants	**Target Region (rsID)**	*PCR Product Lengths*	*Genotype Distributions Product Lengths*
MMP1* *(-1607 1G/2G)	Promotor (rs1799750)	113bp	***1G/1G genotype:*** 102bp, 11bp<br>***1G/2G genotype:*** 113bp, 102bp, 11bp<br>***2G/2G genotype:*** 113bp, 11bp<br>(11bp is not observed).
MMP2* *(-1306 C>T)	Promotor (rs243865)	188bp	***TT genotype:*** 162bp, 26bp, 5bp<br>***CT genotype:*** 188bp, 162bp, 26bp, 5bp<br>***CC genotype:*** 188bp, 5bp<br>(26bp and 5bp are not observed).
MMP2* *(-735 C>T)	Promotor (rs2285053)	391bp	***TT genotype:*** 338bp, 53bp<br>***CT genotype:*** 391bp, 338bp, 53bp<br>***CC genotype:*** 391bp<br>(53bp is not observed).
MMP3* *(-1171 5A/6A)	Promotor (rs3025058)	129bp	***5A/5A genotype:*** 96bp<br>***5A/6A genotype:*** 129bp, 96bp<br>***6A/6A genotype:*** 129bp

Genetic Variants	*PCR Components*	*RFLP Components*
MMP1* *rs1799750* *(-1607 1G/2G)*<br>*MMP2* *rs243865* *(-1306 C>T)*<br>*MMP2* *rs2285053* *(-735 C>T)*<br>*MMP3* *rs3025058* *(-1171 5A/6A)	50 nanograms of isolated DNA<br>0.2 mM dNTP for each<br>forward primer and reverse primer<br>1 x PCR buffer<br>3mM $MgCl_2$<br>1.25 Unite Taq DNA polymerase	1 x Buffer Tango<br>PCR reaction products<br>dH_2O<br>5 Units of restriction enzyme
