# Supplementary material for: Genetic Risk Factors of Neurodegeneration: The Role of MMP1 rs1799750 (−1607 1G/2G), MMP2 [rs243865 (−1306 C > T), rs2285053 (−735 C > T), and MMP3 rs3025058 (−1171 5A/6A) Genetic Variants in Multiple Sclerosis Pathogenesis
Source: J Clin Med. 2026 Oct 2;15(19):7648. doi: 10.3390/jcm15197648 (PMC13665806; doi:10.3390/jcm15197648)
Supplement: Supplementary file 1 [file jcm-15-07648-s001.zip › MS Supplemental File S3.pdf]

Supplemental File S3. Comparison of clinical and demographic parameters between patients with multiple sclerosis and healthy controls

Clinical and demographic parameters	*Patient group (n=100)*	*Control group (n=100)*	*OR (95% Confidence Interval)*	*p*
Age (Mean ± SD)	47.610 ± 12.542	48.130 ± 8.810	0.520 (2.503-3.543)	0.735[a]
Sex (Male / Female)	32 (32%) / 68 (68%)	36 (36%) / 64 (64%)	0.837 (0.466-1.503)<br>1.195 (0.665-2.146)	0.551[b]
Hypertension (+)	18 (18.0%)	17 (17.0%)	1.072 (0.517-2.223)	0.852[b]
Diabetes Mellitus (+)	11 (11.0%)	10 (10.0%)	1.112 (0.450-2.750)	0.818[b]
Cholesterol (+)	13 (13.0%)	8 (8.0%)	1.718 (0.679-4.348)	0.253[b]
Heart Disease (+)	13 (13.0%)	4 (4.0%)	3.586 (1.127-11.413)	***0.031***[b*]
Alcohol (+)	29 (29.0%)	14 (14.0%)	2.509 (1.232-5.109)	***0.011***[b*]
Smoking (+)	35 (35.0%)	21 (21.0%)	2.026 (1.076-3.814)	***0.029***[b*]

[a]Independent Samples Test

[b]Logistic Regression, Chi-square test

SD: Standard Deviation; (+): Available; *: Significance (p<0,05)
