# Supplementary material for: Genetic Risk Factors of Neurodegeneration: The Role of MMP1 rs1799750 (−1607 1G/2G), MMP2 [rs243865 (−1306 C > T), rs2285053 (−735 C > T), and MMP3 rs3025058 (−1171 5A/6A) Genetic Variants in Multiple Sclerosis Pathogenesis
Source: J Clin Med. 2026 Oct 2;15(19):7648. doi: 10.3390/jcm15197648 (PMC13665806; doi:10.3390/jcm15197648)
Supplement: Supplementary file 1 [file jcm-15-07648-s001.zip › MS Supplemental File S4.pdf]

Supplemental File S4. *MMP* gene variants genotype distributions according to average attack numbers for multiple sclerosis patient

Genotype distributions		*Patient group (n=100)* *Average Attack Numbers*	*p*
MMP1	1G/1G	4.486 ± 3.302	
rs1799750	1G/2G	4.641 ± 3.320	0.955[a]
(-1607 1G/2G)	2G/2G	4.769 ± 4.430	
MMP2	CC	5.500 ± 3.146	
rs243865	CT	4.976 ± 4.228	0.517[a]
(-1306 C>T)	TT	4.245 ± 3.100	
MMP2	CC	4.673 ± 3.434	
rs2285053	CT	4.683 ± 3.889	0.847[a]
(-735 C>T)	TT	3.857 ± 3.436	
MMP3	5A/5A	3.231 ± 3.032	
rs3025058	5A/6A	4.326 ± 3.367	0.126[a]
(-1171 5A/6A)	6A/6A	5.390 ± 3.898	

[a]One-way Anova test

CC: Cytosine-Cytosine; **CT:** Cytosine-Thymine; **TT:** Thymine-Thymine; **1G/1G:** 1Guanine/1Guanine; **1G/2G:** 1Guanine/2Guanine; **2G/2G:** 2Guanine/2Guanine; **5A/5A:** 5Adenine/5Adenine; **5A/6A:** 5Adenine/6Adenine; **6A/6A:** 6Adenine/6Adenine

[*]Significance (p<0,05)
