# Supplementary material for: Genetic Risk Factors of Neurodegeneration: The Role of MMP1 rs1799750 (−1607 1G/2G), MMP2 [rs243865 (−1306 C > T), rs2285053 (−735 C > T), and MMP3 rs3025058 (−1171 5A/6A) Genetic Variants in Multiple Sclerosis Pathogenesis
Source: J Clin Med. 2026 Oct 2;15(19):7648. doi: 10.3390/jcm15197648 (PMC13665806; doi:10.3390/jcm15197648)
Supplement: Supplementary file 1 [file jcm-15-07648-s001.zip › MS Supplemental File S5.pdf]

Supplemental File S5. Haplotype analysis according to average attack numbers in patients with multiple sclerosis

Haplotype analysis MMP1 rs1799750/ MMP2 rs243865 (-1306 C>T)	*Patients (N=100)*	*Frequency (%)*	*Average Attack Numbers (Mean ± SD)*	*p*
1G/1G-CC	3	***3.0***	6.333 ± 2.887	
1G/1G-CT	13	***13.0***	4.077 ± 2.985	
1G/1G-TT	19	***19.0***	4.474 ± 3.611	
1G/2G-CT	17	***17.0***	4.588 ± 3.624	0.506[a]
1G/2G-TT	21	***21.0***	4.476 ± 3.076	
2G/2G-CC	2	***2.0***	2.500 ± 0.707	
2G/2G-CT	11	***11.0***	6.636 ± 5.954	
2G/2G-TT	13	***13.0***	3.538 ± 2.367	
Haplotype analysis MMP1 rs1799750/ MMP2 rs2285053 (-735 C>T)	***Patients (N=100)***	***Frequency (%)***	***Average Attack Numbers (Mean ± SD)***	***p***
1G/1G-CC	16	***16.0***	5.188 ± 3.468	
1G/1G-CT	18	***18.0***	4.000 ± 3.298	
1G/2G-CC	20	***20.0***	5.000 ± 3.240	
1G/2G-CT	16	***16.0***	4.625 ± 3.442	0.635[a]
2G/2G-CC	15	***15.0***	3.667 ± 3.677	
2G/2G-CT	8	***8.0***	6.250 ± 5.676	
2G/2G-TT	3	***3.0***	6.333 ± 4.041	
Haplotype analysis MMP1 rs1799750/ MMP3 rs3025058	***Patients (N=100)***	***Frequency (%)***	***Average Attack Numbers (Mean ± SD)***	***p***
1G/1G-5A/5A	7	***7.0***	3.286 ± 3.638	
1G/1G-5A/6A	13	***13.0***	4.308 ± 2.323	
1G/1G-6A/6A	15	***15.0***	5.200 ± 3.858	
1G/2G-5A/5A	3	***3.0***	2.667 ± 2.517	
1G/2G-5A/6A	21	***21.0***	5.048 ± 3.442	0.216[a]
1G/2G-6A/6A	16	***16.0***	4.750 ± 3.088	
2G/2G-5A/5A	2	***2.0***	3.667 ± 2.887	
2G/2G-5A/6A	13	***13.0***	3.538 ± 3.886	
2G/2G-6A/6A	10	***10.0***	7.778 ± 5.674	
Haplotype analysis MMP2 rs243865 (-1306 C>T) / MMP2 rs2285053 (-735 C>T)	***Patients (N=100)***	***Frequency (%)***	***Average Attack Numbers (Mean ± SD)***	***p***

CC-CC	3	***3.0***	6.667 ± 3.215	
CC-CT	3	***3.0***	5.000 ± 3.000	
CT-CC	19	***19.0***	5.211 ± 3.994	
CT-CT	16	***16.0***	6.063 ± 5.893	0.687[a]
TT-CC	28	***28.0***	4.357 ± 3.021	
TT-CT	23	***23.0***	4.130 ± 3.266	
TT-TT	2	***2.0***	6.500 ± 0.707	
Haplotype analysis MMP2 rs243865 (-1306 C>T) / MMP3 rs3025058	***Patients (N=100)***	***Frequency (%)***	***Average Attack Numbers (Mean ± SD)***	***p***
CC-5A/5A	2	***2.0***	2.500 ± 0.707	
CC-5A/6A	2	***2.0***	4.000 ± 1.414	
CC-6A/6A	3	***3.0***	8.333 ± 0.577	
CT-5A/5A	2	***2.0***	1.500 ± 2.121	0.415[a]
CT-5A/6A	24	***24.0***	5.125 ± 4.839	
CT-6A/6A	16	***16.0***	6.250 ± 4.726	
TT-5A/6A	20	***20.0***	4.550 ± 3.052	
TT-6A/6A	22	***22.0***	4.364 ± 3.170	
Haplotype analysis MMP2 rs2285053 (-735 C>T) / MMP3 rs3025058	***Patients (N=100)***	***Frequency (%)***	***Average Attack Numbers (Mean ± SD)***	***p***
CC-5A/5A	5	***5.0***	2.400 ± 1.817	
CC-5A/6A	26	***26.0***	4.385 ± 2.994	
CC-6A/6A	21	***21.0***	6.143 ± 4.442	
CT-5A/5A	6	***6.0***	3.333 ± 3.983	0.311[a]
CT-5A/6A	17	***17.0***	5.647 ± 5.499	
CT-6A/6A	18	***18.0***	5.222 ± 3.735	
TT-5A/6A	3	***3.0***	2.333 ± 0.577	

[a]One-way Anova test; **SD:** Standard Deviation

CC: Cytosine-Cytosine; **CT:** Cytosine-Thymine; **TT:** Thymine-Thymine; **1G/1G:** 1Guanine/1Guanine; **1G/2G:** 1Guanine/2Guanine; **2G/2G:** 2Guanine/2Guanine; **5A/5A:** 5Adenine/5Adenine; **5A/6A:** 5Adenine/6Adenine; **6A/6A:** 6Adenine/6Adenine; [*]Significance (p<0,05)
