# Supplementary material for: Genetic Risk Factors of Neurodegeneration: The Role of MMP1 rs1799750 (−1607 1G/2G), MMP2 [rs243865 (−1306 C > T), rs2285053 (−735 C > T), and MMP3 rs3025058 (−1171 5A/6A) Genetic Variants in Multiple Sclerosis Pathogenesis
Source: J Clin Med. 2026 Oct 2;15(19):7648. doi: 10.3390/jcm15197648 (PMC13665806; doi:10.3390/jcm15197648)
Supplement: Supplementary file 1 [file jcm-15-07648-s001.zip › MS Supplemental File S6.pdf]

**Supplemental File S6.** *MMP* gene variants genotype distributions according to MS duration for multiple sclerosis patient

| *Genotype distributions* | | *Patient group (n=100)* *MS Duration (Year)* | *p* |
|---|---|---|---|
| ***MMP1*** | 1G/1G | 11.800 ± 7.680 | |
| ***rs1799750*** | 1G/2G | 13.154 ± 7.415 | 0.287[a] |
| ***(-1607 1G/2G)*** | 2G/2G | 10.115 ± 7.602 | |
| ***MMP2*** | CC | 9.500 ± 3.987 | |
| ***rs243865*** | CT | 13.537 ± 7.678 | 0.178[a] |
| ***(-1306 C>T)*** | TT | 10.887 ± 7.655 | |
| ***MMP2*** | CC | 12.365 ± 7.441 | |
| ***rs2285053*** | CT | 10.683 ± 7.643 | 0.252[a] |
| ***(-735 C>T)*** | TT | 15.429 ± 7.786 | |
| ***MMP3*** | 5A/5A | 9.615 ± 6.983 | |
| ***rs3025058*** | 5A/6A | 11.156 ± 7.562 | 0.201[a] |
| ***(-1171 5A/6A)*** | 6A/6A | 13.381 ± 7.648 | |

[a]One-way Anova test

**CC:** Cytosine-Cytosine; **CT:** Cytosine-Thymine; **TT:** Thymine-Thymine; **1G/1G:** 1Guanine/1Guanine; **1G/2G:** 1Guanine/2Guanine; **2G/2G:** 2Guanine/2Guanine; **5A/5A:** 5Adenine/5Adenine; **5A/6A:** 5Adenine/6Adenine; **6A/6A:** 6Adenine/6Adenine; [*]Significance (p<0,05)
