# Supplementary material for: Genetic Risk Factors of Neurodegeneration: The Role of MMP1 rs1799750 (−1607 1G/2G), MMP2 [rs243865 (−1306 C > T), rs2285053 (−735 C > T), and MMP3 rs3025058 (−1171 5A/6A) Genetic Variants in Multiple Sclerosis Pathogenesis
Source: J Clin Med. 2026 Oct 2;15(19):7648. doi: 10.3390/jcm15197648 (PMC13665806; doi:10.3390/jcm15197648)
Supplement: Supplementary file 1 [file jcm-15-07648-s001.zip › MS Supplemental File S7.pdf]

Supplemental File S7. Haplotype analysis according to MS duration in patients with multiple sclerosis

Haplotype analysis MMP1 rs1799750/ MMP2 rs243865 (-1306 C>T)	*Patients (N=100)*	*Frequency (%)*	*MS Duration (Year) (Mean ± SD)*	*p*
1G/1G-CC	3	***3.0***	12.333 ± 2.517	
1G/1G-CT	13	***13.0***	13.154 ± 7.883	
1G/1G-TT	19	***19.0***	10.789 ± 8.176	
1G/2G-CT	17	***17.0***	13.765 ± 7.319	0.357
1G/2G-TT	21	***21.0***	13.000 ± 7.662	
2G/2G-CC	2	***2.0***	7.000 ± 4.243	
2G/2G-CT	11	***11.0***	13.636 ± 8.675	
2G/2G-TT	13	***13.0***	7.615 ± 6.035	
Haplotype analysis MMP1 rs1799750/ MMP2 rs2285053 (-735 C>T)	***Patients (N=100)***	***Frequency (%)***	***MS Duration (Year) (Mean ± SD)***	***p***
1G/1G-CC	16	***16.0***	12.000 ± 7.581	
1G/1G-CT	18	***18.0***	10.278 ± 6.875	
1G/2G-CC	20	***20.0***	14.800 ± 7.135	
1G/2G-CT	16	***16.0***	12.063 ± 7.937	0.265[a]
2G/2G-CC	15	***15.0***	9.933 ± 7.294	
2G/2G-CT	8	***8.0***	8.250 ± 8.697	
2G/2G-TT	3	***3.0***	16.000 ± 4.359	
Haplotype analysis MMP1 rs1799750/ MMP3 rs3025058	***Patients (N=100)***	***Frequency (%)***	***MS Duration (Year) (Mean ± SD)***	***p***
1G/1G-5A/5A	7	***7.0***	8.143 ± 7.403	
1G/1G-5A/6A	13	***13.0***	12.846 ± 8.081	
1G/1G-6A/6A	15	***15.0***	12.600 ± 7.443	
1G/2G-5A/5A	3	***3.0***	12.667 ± 7.371	
1G/2G-5A/6A	21	***21.0***	12.786 ± 7.424	0.264[a]
1G/2G-6A/6A	16	***16.0***	14.563 ± 7.474	
2G/2G-5A/5A	2	***2.0***	13.000 ± 7.071	
2G/2G-5A/6A	13	***13.0***	7.231 ± 6.405	
2G/2G-6A/6A	10	***10.0***	13.900 ± 8.144	
Haplotype analysis MMP2 rs243865 (-1306 C>T) / MMP2 rs2285053 (-735 C>T)	***Patients (N=100)***	***Frequency (%)***	***MS Duration (Year) (Mean ± SD)***	***p***
CC-CC	3	***3.0***	10.333 ± 4.509	

CC-CT	3	***3.0***	7.667 ± 3.215	
CT-CC	19	***19.0***	13.474 ± 7.633	
CT-CT	16	***16.0***	12.438 ± 7.536	0.709[a]
TT-CC	28	***28.0***	12.071 ± 7.874	
TT-CT	23	***23.0***	9.783 ± 7.897	
TT-TT	2	***2.0***	13.500 ± 6.364	
Haplotype analysis MMP2 rs243865 (-1306 C>T) / MMP3 rs3025058	***Patients (N=100)***	***Frequency (%)***	***MS Duration (Year) (Mean ± SD)***	***p***
CC-5A/5A	2	***2.0***	8.000 ± 5.657	
CC-5A/6A	2	***2.0***	10.000 ± 0.000	
CC-6A/6A	3	***3.0***	10.333 ± 4.509	
CT-5A/5A	2	***2.0***	1.000 ± 0.000	0.126[a]
CT-5A/6A	24	***24.0***	12.500 ± 7.757	
CT-6A/6A	16	***16.0***	15.750 ± 6.807	
TT-5A/6A	20	***20.0***	9.600 ± 7.394	
TT-6A/6A	22	***22.0***	12.545 ± 8.040	
Haplotype analysis MMP2 rs2285053 (-735 C>T) / MMP3 rs3025058	***Patients (N=100)***	***Frequency (%)***	***MS Duration (Year) (Mean ± SD)***	***p***
CC-5A/5A	5	***5.0***	11.400 ± 7.829	
CC-5A/6A	26	***26.0***	12.731 ± 6.803	
CC-6A/6A	21	***21.0***	14.095 ± 7.628	
CT-5A/5A	6	***6.0***	6.333 ± 6.055	0.207[a]
CT-5A/6A	17	***17.0***	9.882 ± 7.193	
CT-6A/6A	18	***18.0***	12.222 ± 7.878	
TT-5A/6A	3	***3.0***	17.667 ± 10.693	

[a]One-way Anova test; **SD:** Standard Deviation

CC: Cytosine-Cytosine; **CT:** Cytosine-Thymine; **TT:** Thymine-Thymine; **1G/1G:** 1Guanine/1Guanine; **1G/2G:** 1Guanine/2Guanine; **2G/2G:** 2Guanine/2Guanine; **5A/5A:** 5Adenine/5Adenine; **5A/6A:** 5Adenine/6Adenine; **6A/6A:** 6Adenine/6Adenine; [*]Significance ($p<0,05$)
