# Supplementary material for: Genetic Risk Factors of Neurodegeneration: The Role of MMP1 rs1799750 (−1607 1G/2G), MMP2 [rs243865 (−1306 C > T), rs2285053 (−735 C > T), and MMP3 rs3025058 (−1171 5A/6A) Genetic Variants in Multiple Sclerosis Pathogenesis
Source: J Clin Med. 2026 Oct 2;15(19):7648. doi: 10.3390/jcm15197648 (PMC13665806; doi:10.3390/jcm15197648)
Supplement: Supplementary file 1 [file jcm-15-07648-s001.zip › MS Supplemental File S8.pdf]

**Supplemental File S8.** Model Performance and Nagelkerke $R^2$ Change Statistics

| **Model / Included Variables** | **Nagelkerke $R^2$** | **$R^2$ Change ($\Delta R^2$)** | **Interpretation/ Explanatory Power** |
|---|---|---|---|
| **Baseline Model** *(Age, sex, HT, DM, cholesterol, heart disease, alcohol, smoking)* | 0.082 | — | Baseline demographic and clinical factors |
| Baseline + *MMP1* rs1799750 (-1607 1G/2G) | 0.095 | +0.013 | Slight improvement |
| Baseline + *MMP2* rs243865 (-1306 C>T) | 0.284 | +0.202 | **Substantial improvement (High utility)** |
| Baseline + *MMP2* rs2285053 (-735 C>T) | 0.112 | +0.030 | Moderate improvement |
| Baseline + *MMP3* rs3025058 (-1171 5A/6A) | 0.128 | +0.046 | Moderate improvement |

Nagelkerke $R^2$ values were derived from binary logistic regression models to assess the added explanatory power of each genetic variant beyond traditional baseline factors. ΔR2 represents the increase in model performance relative to the baseline clinical model.
